# F_1_-ATPase Rotary Mechanism: Interpreting Results of Diverse Experimental Modes With an Elastic Coupling Theory

**DOI:** 10.3389/fmicb.2022.861855

**Published:** 2022-04-22

**Authors:** Sándor Volkán-Kacsó, Rudolph A. Marcus

**Affiliations:** ^1^Noyes Laboratory of Chemical Physics, California Institute of Technology, Pasadena, CA, United States; ^2^Segerstrom Science Center, Azusa Pacific University, Azusa, CA, United States

**Keywords:** F1-ATPase, stepping rotation of F1-ATPase, rotary biomolecular motors, cryo-electron microscopy, single-molecule imaging, multi-state theory, ATP binding, concerted kinetics

## Abstract

In this chapter, we review single-molecule observations of rotary motors, focusing on the general theme that their mechanical motion proceeds in substeps with each substep described by an angle-dependent rate constant. In the molecular machine F1-ATPase, the stepping rotation is described for individual steps by forward and back reaction rate constants, some of which depend strongly on the rotation angle. The rotation of a central shaft is typically monitored by an optical probe. We review our recent work on the theory for the angle-dependent rate constants built to treat a variety of single-molecule and ensemble experiments on the F_1_-ATPase, and relating the free energy of activation of a step to the standard free energy of reaction for that step. This theory, an elastic molecular transfer theory, provides a framework for a multistate model and includes the probe used in single-molecule imaging and magnetic manipulation experiments. Several examples of its application are the following: (a) treatment of the angle-dependent rate constants in stalling experiments, (b) use of the model to enhance the time resolution of the single-molecule imaging apparatus and to detect short-lived states with a microsecond lifetime, states hidden by the fluctuations of the imaging probe, (c) treatment of out-of-equilibrium “controlled rotation” experiments, (d) use of the model to predict, without adjustable parameters, the angle-dependent rate constants of nucleotide binding and release, using data from other experiments, and (e) insights obtained from correlation of kinetic and cryo-EM structural data. It is also noted that in the case where the release of ADP would be a bottleneck process, the binding of ATP to another site acts to accelerate the release by 5–6 orders of magnitude. The relation of the present set of studies to previous and current theoretical work in the field is described. An overall goal is to gain mechanistic insight into the biological function in relation to structure.

## Introduction

Fueled by the free energy of ATP hydrolysis, cellular nanomachinery works to realize functions, such as muscle contraction, cell motility, cell division, intracellular protein transport, wound healing, and DNA repair (McLaughlin et al., 2016; Saper and Hess, 2020 and references cited therein). These nanomachines are rotary motors (F- or V-ATPases), linear motors (myosins, kinesins, and dyneins), and revolving motors (viral packaging motors and DNA motor complexes; Guo et al., 2016; Tong and Bustamante, 2021), in which biological function arises from the interconversion of chemical free energy of ATP (or in some machines, GTP), hydrolysis/synthesis, and mechanical energy of conformational changes (Braig et al., 2000; Senior, 2007; Weber, 2010; Mukherjee and Warshel, 2011, 2015; Junge and Nelson, 2015). Single-molecule imaging is used to resolve the kinetic steps in relation to mechanical displacement, which is also stepwise (Noji et al., 1997; Yasuda et al., 2001; Nishizaka et al., 2004; Sakaki et al., 2005; Adachi et al., 2007, 2012; Sielaff et al., 2008, 2016; Spetzler et al., 2009; Watanabe et al., 2010; Sugawa et al., 2016; Miller et al., 2018).

**Figure 1:**
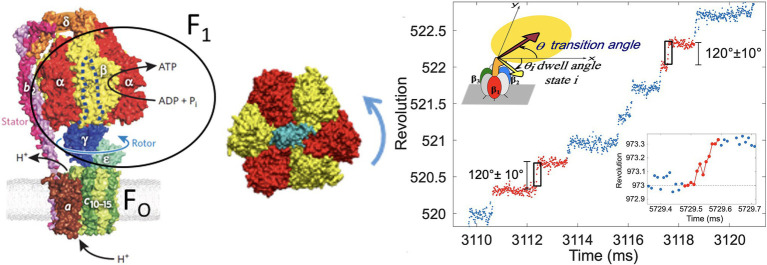
F-ATPase structure showing the F1 and Fo components (left). F1-ATPase motor “transverse cross-section” view showing the central rotor in blue and α ring subunits in gold and β subunits in red (center). Example of a trajectory showing stepping rotation of F1-ATPase (right), as imaged by a probe in single molecule experiment (top inset). A transition is shown (red points) between two dwells (blue dots) in the lower inset. Figures were reproduced, with permission from Weber (2010) and Volkán-Kacsó et al. (2019).

In particular, the F-ATPase (ATP synthase) uses a proton concentration gradient across the mitochondrial membrane (or cell membrane in prokaryotes) to synthesize ATP from ADP and inorganic phosphate (Pi), an energetically uphill process, *via* a rotating shaft (Boyer, 1993; Weber and Senior, 1997), seen in the structure from Figure 1 (Braig et al., 2000). F_1_-ATPase is the water-soluble part of the F-ATPase, and it is the minimal subsystem that functions as a rotary motor. Single-molecule imaging experiments (Iino and Noji, 2013) in the Thermophilic Bacillus F_1_-ATPase demonstrated that it converts chemical free energy from ATP hydrolysis into a stepping rotation of 80 and 40° substeps by a coordinated mechanism (Senior et al., 2002).

The stepping rotation of a single F_1_-ATPase motor enzyme (Figure 1) proceeds on a timescale of milliseconds (or, under certain conditions, seconds), while the fluctuations during the transitions relax within micro- to nanoseconds. The stepping behavior involves a concerted mechanism involving a small molecule and the host subunits, which undergo conformational dynamics, driving the directional mechanical motion (e.g., rotation) and leading to the biological functions. The microsecond processes, present in F_1_-ATPase and across all types of motors, have eluded detection in single-molecule imaging experiments and was only recently captured by atomistic computer simulations in kinesin (Hwang et al., 2017).

We review some recent theoretical works treating microsecond to second kinetics from single motors, at high and low ATP concentrations, applied to −F_1_-ATPase from *Thermophilic Bacillus* (ThF1) and *Paracoccus denitrificans* (PdF1) in relation to their structure. The topics in the review are as follows: In the section “Relevance of single-molecule F1-ATPase experiments and theory to operation of FOF1 ATP synthase,” we provide some arguments supporting the relevance for the function of the F-ATP synthase of the single-molecule experiments and theory. In the section “Use of data on ensemble studies to assist in predictions of single-molecule measurements,” we review an elastic transfer theory of F_1_-ATPases and its predictions of stalling experiments. In the section “Predictions of angle-dependent rate constants for binding and release in ThF1, using controlled rotation data at Low ATP concentrations,” the use of theory to predict rate constants in slow, low ATP concentration, controlled rotation experiments in ThF1 is reviewed. In the section “Detection of an intermediate state related to ATP binding and ADP release, observed at high ATP concentrations,” a related method using velocities to extract fast states in ThF1 is described. In the section “Behavior of the system in the dwell region using statistical methods and the forced diffusion equation to study the statistical mechanical properties,” recent work on applying a velocity method to PdF1 to study shorter time scales is reviewed. In the section “Comparison of kinetics and the structural behavior inferred from cryo-EM studies,” the implications of the single-molecule elastic transfer theory for the kinetics-structure relation are discussed, with emphasis on recent Cryo-EM structures. In the section “Relation between mechanical motion and chemistry,” a relation between single-molecule kinetic and chemistry is discussed from studies at low ATP concentration. In the section “Relation to other theoretical and computational work,” our results are related to other theoretical and computational work in the field of single-molecule imaging of biomolecular motors.

## Relevance of Single-Molecule F_1_-ATPase Experiments and Theory to Operation of F_O_F_1_ ATP Synthase

The complete enzyme, F_O_F_1_ ATP Synthase, contains a number of subunits not present in single-molecule F_1_-ATPase studies. In particular, it contains the in-membrane F_o_ assembly that includes the c subunits as well as a δ and ϵ subunit. In some bacteria, the ϵ subunit acts as a rachet to reduce the possibility of a back-reaction (García-Trejo, and Morales-Ríos, 2008), while in other systems like the mammalian F-ATPase the δ and ϵ subunits are incorporated into the globular section of the rotor shaft and they do not work as ratchets. The question naturally arises of the relevance experimental and theoretical studies of this truncated system, F_1_-ATPase, which is the operation of the F_O_F_1_ system. A ratchet, for example, is not needed if the forward reaction is very downhill, and so is sometime operative and sometimes not.

The full F_0_F_1_ system also uses an acid–base reaction at the beginning and at the end of an offset channel in the F_O_ unit to cause a rotation of a central γ shaft in the F_1_ (Bai and Warshel, 2019; Sielaff et al., 2019). In the single-molecule experiments, this rotation is accomplished instead by externally controlled rotation of the γ shaft (Adachi et al., 2000) or by performing stalling (Watanabe et al., 2012) or free rotation experiments (Adachi et al., 2007).

The successive proton jumps from position in the entrance channel to a (Bai and Warshel, 2019) carboxyl group in the nearest *c*-ring member causing a rotation to occur because it is sufficiently downhill or because the ratchet is used to inhibit the back reaction. Thereby, we have in the F_O_-ATPase a stepped rotation. This stepped rotation is paralleled in the single-molecule controlled rotation experiments by collecting data on the occupation of successive cells in angle space of a size comparable to 360°/*N*, where *N* is the number of *c* subunits in the F_O_ portion of the F_O_F_1_ ATP synthase. So, in this respect, these single-molecule experiments simulate the stepwise motion of the gamma shaft caused by the stepped acid base proton transfer reaction and their behavior. In this aspect, it is reasonable to infer that the single-molecule experiments in these controlled rotation experiments are relevant to those to the full enzyme. Inasmuch as the stalling experiments can be used to predict the controlled rotation experiment, as discussed in the section “Use of data on ensemble studies to assist in predictions of single-molecule measurements,” in obtaining agreement with no adjustable parameters, the implication is that the stalling experiments are also relevant to providing insight into the kinetic behavior and parameters of the F_O_F_1_ ATP Synthase.

## Use of Data on Ensemble Studies to Assist in Predictions of Single-Molecule Measurements

**Scheme 1:**
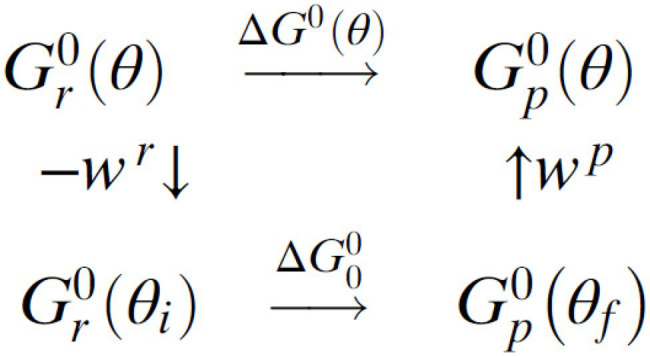
Thermodynamic cycle for the nucleotide binding step.

Using ideas that originated in the 1960s in the transfer of atoms and groups, we applied them (Volkán-Kacsó and Marcus, 2015, 2016, 2017a) to the binding and release of ATP in single-molecule experiments in which the rotation angle is stalled or rotated at a constant angular velocity. In F1-ATPase, the dwell angles are angles of local stability and so they serve to identify initial and final states during a transition from one dwell to the next. A thermodynamic cycle (Scheme 1) provides a relationship between the free energies of a nucleotide binding when the angle of the rotor shaft is not constrained by the imaging probe, $\Delta G_{0}^{0}$ , and the binding free energy ∆*G^0^(θ)* at a constant rotor angle *θ*. If the free energies in the reactant and product spaces are denoted using indices r and p , and the stable dwell angles in these states by $\theta_{i}$ and $\theta_{f}$ , the thermodynamic cycle is:

Due to an elastic coupling of the probe to the stator ring that includes the rotor shaft (Sielaff et al., 2008), according to Scheme 1, keeping the system at a fixed angle θ would require an elastic energy, both when the system is in the reactant and product states,


(1) $$w^{r}=\frac{k}{2}{\left(\theta -\theta_{i}\right)}^{2}\;\mathrm{and}\;w^{p}=\frac{k}{2}{\left(\theta -\theta_{f}\right)}^{2}$$

Then, for the standard free energy of reaction it is found that (Volkán-Kacsó and Marcus, 2015),


(2) $$\begin{array}{l} \Delta G^{0}\theta =\Delta G_{0}^{0}+w^{p}\;\theta -w^{r}\theta \\ \;=\Delta G_{0}^{0}-k\;\theta_{f}-\theta_{i}\left[\theta -\theta_{f}+\theta_{i}/2\right] \end{array}$$

The elastic terms for the reactants and products of an individual step in a subunit are given as a function of *θ* in equation (1), and, using a parabolic relation for the transfer process (Marcus, 1968, 1993; Marcus and Sutin, 1985), they yield an equation for the free energy barrier as a function of *θ*,


(3) $$\Delta G*\left(\theta \right)=W^{r}+{\left[\lambda +\Delta G^{0}\left(\theta \right)\right]}^{2}/4\lambda$$

**Figure 2:**
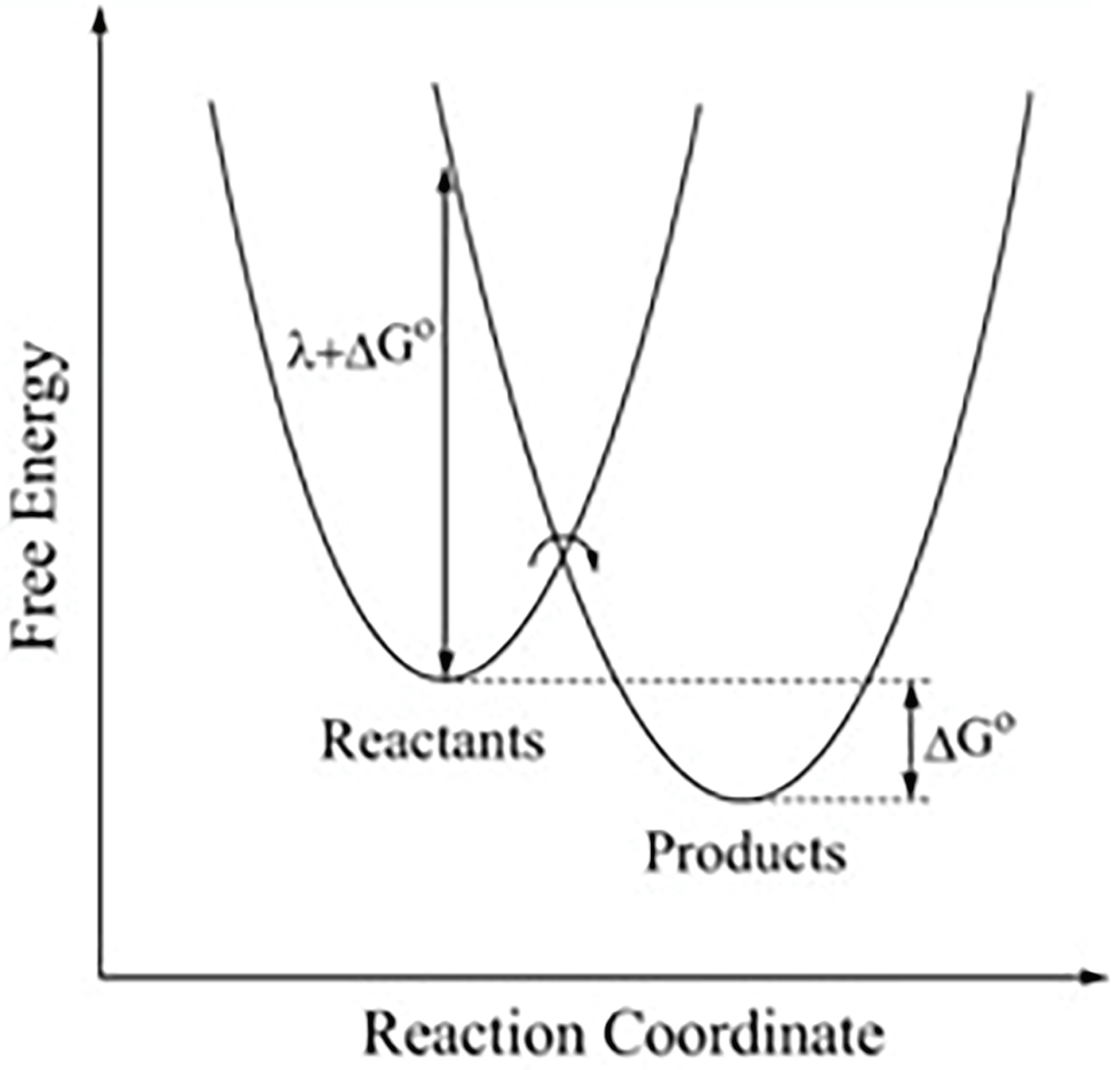
Free energy curves for reactant and product states showing the definition of $\Delta G^{0}$ and λ . These two parabolas are plotted vs. the reaction coordinate that describes the progress of the reaction. The transition state occurs at the intersection of the two parabolas, as also in Figure 1 in Marcus and Sutin (1985). For simplicity of presentation, some symbols and the labeling of work terms are omitted here.

A work term *W^r^* arises from the attachment of the ATP from solution to the exterior of the F_1_-ATPase, and the standard free energy of reaction ∆*G^0^(θ)* is given as a function of *θ* in equation (2) (Volkán-Kacsó and Marcus, 2015). Stalling experiments provide the *θ*-dependent rate constants and equilibrium constants for the steps. The ∆*G*^0^(*θ*) is related to the *θ*-dependent equilibrium constant *K(θ)* for that step by ∆*G^0^*(*θ*) = − *kT* ln *K*(θ) and so ln *K*(θ) for ATP binding/release is linear in θ, a result also seen in the experimental data in Figure 2 (noting that the “*k*” in this equation is the Boltzmann constant). In passing, we note that equation (3), without the θ, has also been extensively applied to atom, ion, and group transfer reactions (Wolfe and Mitchell, 1981; Lewis and Hu, 1984; Lewis, 1986; Warren and Mayer, 2012).

We consider next the physical meaning of λ appearing in equations (2) and (3). The standard theory of chemical reaction rates, transition state theory (e.g., Glasstone et al., 1941; Truhlar et al., 1996), expresses the reaction rate constant in terms of a free energy barrier, which in the present application is given by equations (2) and (3) for each value of the rotation angle, θ . This free energy barrier to reaction depends on how downhill or uphill the reaction is, namely it depends on ∆*G*°. It also depends on what is known as the reorganization energy, λ , in the electron transfer literature and depends on the work term *w*^r^ to bring the system from the dwell angle of the reactants $\theta_{i}$ to the angle θ , followed by the various internal changes to reach the transition state at that angle, and also on the work term *w*^p^ associated with reaching the dwell angle $\theta_{f}$ .

In Figure 2, *λ* is seen there to be the vertical difference of the products’ and the reactants’ free energy evaluated at the equilibrium distribution of coordinates of the reactants and their environment and corrected for the contribution of ∆G° at the given θ. One sees from equations (4–6) of Marcus and Sutin (1985) that *λ* depends on the magnitude of the changes and not on their sign. That is, the breaking of a hydrogen bond contributes the same amount to *λ* as the forming of a hydrogen bond. For example, in the case of an ATP entering a cleft in the ATPase, if one counts the number of hydrogen bonds of the ATP that are broken or formed, they contribute equally to *λ*. So, it will contain the sum of the free energies of these two quantities. Similarly, λ will contain the free energy changes due to other structural changes such as changes in dielectric polarization of the medium surrounding the charges regardless of sign, at any given rotation angle θ. In the particular case of electron transfer reactions (Marcus and Sutin, 1985), equations were given for λ that depends on the size of the reacting ions, the change in ionic charges of each reactant, the resulting changes in solvation, and the changes in bond lengths and angles, all of which accompany an electron transfer. In applying equation (3) for ∆G* to atom or group transfers, we note that they are topologically different from that of electron transfers. A result is that equation (3) is confined to the region | $\Delta G^{0}|/\lambda$ which is equal to or less than unity, outside a range termed the inverted region in electron transfer (Marcus and Sutin, 1985). In passing, we note that the quantity *λ* plays an important role in interpreting in physical chemical terms the extensive experimental data on the rates of electron transfer reactions. The ideas were further extended to bond breaking—bond-forming reactions, using a bond energy-bond order model (Marcus, 1968).

All of these remarks refer to the case where transition state theory and its quasi-equilibrium assumption for the formation of the transition state are applicable. There are some situations, particularly for some reactions that are ultrafast, where the rate constant depends also on a slow relaxation time of some key coordinate in adjusting to the new configurations. In this case, transition state theory, which assumes instead a quasi-equilibrium between the transition state and the reactants, requires modification. Details are given in the famous paper by Kramers in 1940 for systems treated one dimensionally (Kramers, 1940) and elaborated upon in numerous papers since. An example of an elaboration involves the case where there are two coordinates to be considered, one of which is “fast” and the other of which is “slow” (Agmon and Hopfield, 1983; Sumi and Marcus, 1986) and contributed to by many authors. The applicability of this type of formalism to F_1_-ATPase was suggested in Watanabe et al. (2013).

The Brønsted slope, or its counterpart in electrochemistry, the Tafel slope, is often used to provide mechanistic information on chemical reactions. Under certain conditions (constant *λ*), it gives insight into where the bottleneck to the reaction lies (the “transition state”), for example whether it is close to the reactants configuration (a slope close to zero) or close to the product’s configuration (a slope close to unity) or in between (close to 0.5).

In the present system, the F1-ATPase, the Brønsted slope for ATP addition and ejection are close to 0.5, found both from interpreting stalling experiments (Adachi et al., 2012; Volkán-Kacsó and Marcus, 2015) and from a theoretical prediction using quantities from other independent ensemble and single-molecule experiments. Meanwhile, for the hydrolysis step it is close to unity (Adachi et al., 2012; Volkán-Kacsó and Marcus, 2017b), and correspondingly for the synthesis step it is close to zero, all of which provides mechanistic information.

The angle dependence of the hydrolysis reaction substep indicates, in the theory, elastic coupling of the reaction with conformational changes in the host subunit (Volkán-Kacsó and Marcus, 2017b), yielding a prediction that the hydrolysis substep produces a small rotation of about 15°. Atomistic simulations of the hydrolysis reaction (Dittrich et al., 2004) in the catalytic pocket support the coupling model. This coupling involves of an Arginine “finger,” αR373, which can be displaced, thereby reducing the reaction free energy barrier. The arginine finger is part of the neighboring α subunit, and its displacement appears to be linked to the opening/closing of that α/β interface, as discussed later in the section “Relation to other theoretical and computational work.”

Free energy simulations have been shown (Warshel et al., 1994) to be a promising tool for descriptions of Brønsted slopes of complex systems. We note that in other systems’ dissociations in the gas phase, it was suggested (e.g., Wardlaw and Marcus, 1988) that the unimolecular dissociations often have a transition state with internal degrees of freedom similar to those of the separated products of the dissociating reactant. In Brønsted slope phrasing, these systems would also thereby have a Brønsted slope near unity.

Regarding the Brønsted slopes of unity for the hydrolysis reaction and correspondingly zero for the reverse reaction, ATP synthesis, independent information on the nature of the transition state for the hydrolysis can be obtained when the Michaelis–Menten expression for the rate constant is valid, using data at the high ATP concentration at different temperatures. When the resulting Arrhenius pre-exponential factor is large, much larger than $10^{13}$ s^−1^, say $\sim 10^{16}$ s^−1^, an argument can be given that the transition state’s internal structure represents that of the products (c.f., Wardlaw and Marcus, 1988 and references cited therein) and hence would yield a Brønsted slope of unity. On the other hand, if the data show a pre-exponential factor at high ATP concentration approximately $10^{13}$ s^−1^ one can argue that typically, other things being equal, the transition state’s internal structure likely resembles that of the reactants, and so would correspond to the Brønsted slope of zero. We plan to investigate this issue further using both ensemble and single molecule data.

However, because of solvational effects accompanying the formation of the transition state for the hydrolysis, further analysis is needed. The Arginine finger, mentioned earlier in this section, is displaced into the proximity of the γ phosphate group, in a configuration that stabilizes the free energy barrier for bond cleavage (Dittrich et al., 2004), and its role in the entropy of activation of the reaction and hence in affecting the pre-exponential factor of the hydrolysis needs to be considered. In passing, we note that from a quantitative point of view, if the reaction involves more than one step but if there is nevertheless a single bottleneck, only the standard free energy of that state relative to that of the reactants is needed for computation of the chemical reaction rate and its temperature dependence. Information about any other intermediate states plays no role.

## Predictions of Angle-Dependent Rate Constants for Binding and Release in ThF1, Using Controlled Rotation Data at Low ATP Concentrations

Single-molecule experiments yielded rotation angle-dependent rate constants for ATP binding or Pi release in the ThF1 (Watanabe et al., 2012). Fluorescently labeled ATP and ADP were used to detect individual events of their binding and release. To treat and explain these data, an elastic chemo-mechanical model was proposed (Volkán-Kacsó and Marcus, 2015). In it, the molecular transfer of nucleotides occurs in tandem with a hinge bending or closing motion of the host subunit coupled to the rotation of the shaft.

**Figure 3:**
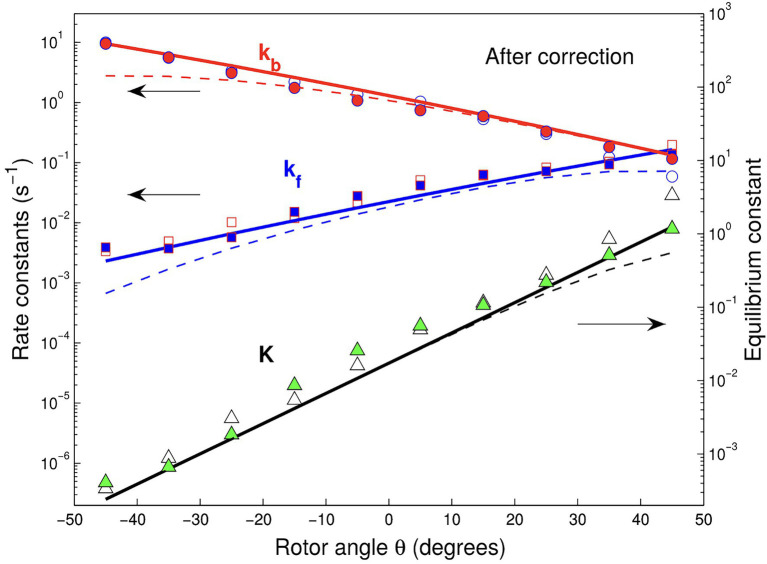
Binding (forward) and release (back) rate and equilibrium rate constants vs. θ angle for Cy3-ATP in the presence (solid squares, circles, and triangles) and absence of Pi (open symbols) in solution. The experimental data of Adachi et al. corrected for missed events are compared with their theoretically predicted counterparts (solid lines). Dashed lines show the data without corrections. The figure is reproduced from Volkán-Kacsó and Marcus (2016).

Subsequently, the theory was used to predict, without adjustable parameters, the exponential angular dependence of ATP binding/release rate constants (Volkán-Kacsó and Marcus, 2016), seen in Figure 3. We note that in the theoretical treatment, the effects of the relatively long-imaging frame times were taken into account by allowing binding/release processes to occur within an imaging frame, resulting in missed events in the single-molecule trajectories.

Controlled rotation experiments performed on the F_1_-ATPase mimic, to some degree, the conditions of rotational catalysis in the whole F_O_F_1_-ATPase, by enforcing the rotation in the ATP synthesis direction. If the enforced rotation is slow compared to the relaxation of the protein structure, the reaction kinetics in the F1-ATPase is described in this quasi-static condition by angle-dependent rate constants, which translate to time-dependent rate constants (Volkán-Kacsó and Marcus, 2016). When the discrete stepping of the Fo part is considered, the compliant structure of the rotor shaft was assumed to have an effect of smoothing out the rotation velocity (Oster and Wang, 2000; Panke et al., 2001); therefore, the framework of steps with angle-dependent rate constants becomes applicable to the rotation of the F_O_F_1_-ATPase.

## Detection of an Intermediate State Related to ATP Binding and ADP Release, Observed at High ATP Concentrations

Recently, the theory was used as a basis for detecting an intermediate state (Volkán-Kacsó et al., 2019). The method relies on the analysis of angular jumps, a measure of the instantaneous velocity of rotation, detected by a nanoscale probe attached to the rotor shaft of the ThF1 motor. The method of using the angle-dependent velocity of the rotation for the analysis of rotation in F_1_-ATPase was first proposed by Frasch and co-workers, who monitored fast single-molecule rotation using polarization spectroscopy with a nanorod probe thereby achieving high temporal resolution (Spetzler et al., 2009). In a recent work, Frasch and coworkers found evidence in single-molecule rotation experiments of a large power-stroke associated with the ADP release. In this section, we discuss our findings (Volkán-Kacsó et al., 2019) using a multistate model of a short-lived state in ThF1, likely before the ADP release. These finding support Frasch’s conclusions, namely that about half of the 80° substep is due to a power stroke provided by the release of ADP, the other half being a result of ATP binding (to another subunit). A multistate model, which is consistent with a previously established rotation scheme (Watanabe et al., 2010) of sequential steps, was utilized. This constraining information was applied (Volkán-Kacsó et al., 2019) to an earlier model (Oster and Wang, 2000) to treat the imaging probe as (1) a Brownian particle of diffusion coefficient D elastically coupled to the stator ring *via* an elastic rotor shaft of spring constant $\kappa_{r}$ , where (2) the potential minimum $\theta_{i}$ could “switch” according to angle-dependent forward and back rate constants, $k_{f}\left(\theta \right)$ and $k_{b}\left(\theta \right)$ . The resulting equations for the angle- and time-dependent probability density $\rho_{i}\left(\theta ,t\right)$ for each state i are (Volkán-Kacsó et al., 2019),


(4) $$\begin{array}{l} \frac{\partial \rho_{i}}{\partial t}=D\frac{\partial }{\partial \theta }\left[\beta \kappa_{r}\;\theta -\theta_{i}\rho_{i}\right]+D\frac{\partial^{2}\rho_{i}}{\partial \theta^{2}}\\ \;-\left[k_{f,i}\;\theta \;+k_{b,i-1}\;\theta \;\right]\rho_{i}+k_{f,i-1}\;\theta \;\rho_{i-1}+k_{b,i}\;\theta \;\rho_{i+1} \end{array}$$

When the motor is found in a given state *I*, it is subject to an elastic restoring force, acting so as to restore the system to the dwell angle of that state. A key quantity used is the jump Δθ in the trajectories, i.e., the change in the angular position from one imaging frame to the next. This jump, when divided by the imaging frame time Δt , yields an estimate for the instantaneous velocity,


(5) $$\upsilon \left(t\right)=\frac{\Delta \theta }{\Delta t}|{}_{t}=\frac{\theta \left(t+\Delta t\right)-\theta \left(t\right)}{\Delta t}$$

**Figure 4:**
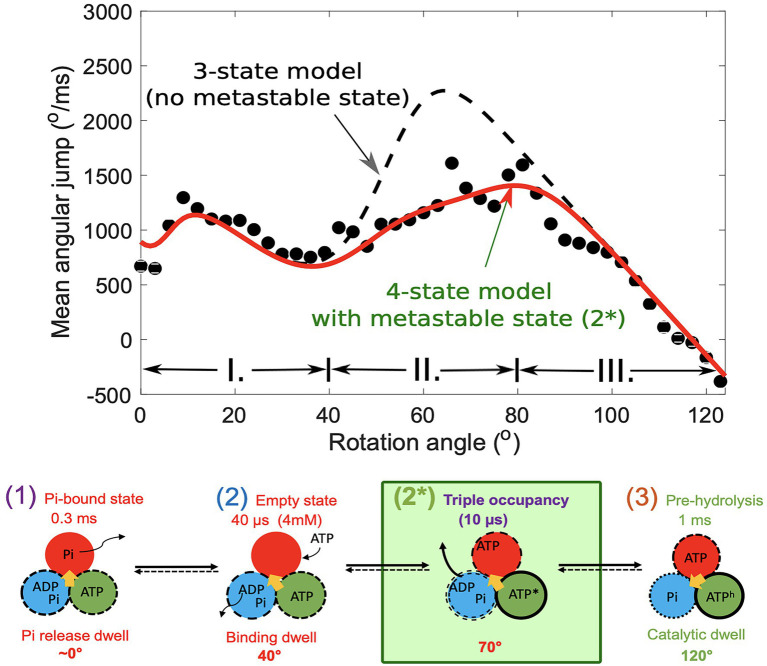
Mean angular jump as a function of rotation angle (top) in fast rotation trajectories. Black dots indicate experimental points, the dashed line shows the predictions from a three-state model, and the continuous line was calculated assuming a fourth state. It is a three-occupancy state. The ATP* and ATP^h^ indicate that the ATP in the pocket is in different environments sue to conformational changes in their host subunits. The figure was reproduced from Volkán-Kacsó et al. (2019).

The mean velocity, now as a function of the rotation angle, $v\left(\theta \right)$ , can be calculated from averaging over jump events in an angular bin. When $v\left(\theta \right)$ is also calculated using a 3- or 4-state model and compared with the experimental velocity (Figure 4), it was found that a fourth state was needed to accurately describe the experiment. In Figure 3, the dashed line shows that a 3-state model would predict a large “hump” in the velocity (or jump) which is not seen in the experimental data. A slow-down of the rotation then is induced by the presence of a short-lived fast state, state 2* in the scheme in Figure 4.

The method thereby revealed a metastable state with a 12-μs lifetime after ATP binding and before ADP release. The detected fast state is similar to a three-occupancy Walker X-ray crystallography structure in which all active sites are occupied by nucleotides. It was concluded that ADP release is accelerated 10,000 times by ATP binding to another subunit, removing a bottleneck that would otherwise prevent the efficient function of the biological motor. We note that this conclusion from theoretical analysis correlates well with results of the original ensemble kinetics experiments that reported such cooperative acceleration (Grubmeyer et al., 1982). Both ATP binding and the induced ADP release are associated with large 40° powerstrokes, which is consistent with conclusion in a recent work by Frasch and coworkers (Martin et al., 2018).

## Behavior of the System in the Dwell Region Using Statistical Methods and the Forced Diffusion Equation to Study the Statistical Mechanical Properties

The spring constant is related to the width of the Brownian fluctuations in the dwells by a simple expression. The SD σ (measured in radians) yields a spring constant in units of pN·nm (Sielaff et al., 2008),

(6) $$\kappa_{m}=4.1/\sigma^{2}$$

where σ is found *via* fitting the histograms with Gaussians. If the relaxation time τ of the system is comparable or faster than the imaging time Δt , the width of the Gaussians is artificially narrowed, so a correction is applicable (Volkán-Kacsó et al., 2019),

(7) $$\kappa_{r}=2\left[\frac{\tau }{\Delta t}-\frac{\tau^{2}}{\Delta t^{2}}\left(1-e^{-\Delta t/\tau }\right)\right]\kappa_{m}\text{.}$$

To utilize dwells from all three subunits in the same analysis, a correction to the trajectories was applied to correct for any tilt of the rotor axis, which yielded a more symmetric set of angular distributions for the 120^o^ steps, seen in Figure 4. Then, the elastic spring constant for ThF1 was found to be about 70 pN nM. In contrast, in recent investigation, we yielded wider fluctuations in PdF1 (Zarco-Zavala et al., 2020) corresponding to a smaller spring constant, in the range of 25–35 pN nM. We note that different trajectories yielded somewhat different values for the spring constant.

The harmonic elastic response of the rotor shaft extends to a remarkably large displacement in PdF1 trajectories, displacements of 60°–70° in both clockwise and counterclockwise directions, as evidenced by linear velocity-angle dependence. In the Brownian regime, the average velocity is proportional to the restoring force; hence, a linear dependence on the rotation angle displacement relative to the dwell angle is indicative of a harmonic spring response. The slope of the velocity line in the dwell agrees with that at the end of the transition, thereby indicating that in the second part of the transition the system has already jumped to the next dwell and the probe is relaxing to the new energy minimum.

A comparison of molecular ratchet vs. power stroke mechanism was discussed in a recent perspective (Hwang and Karplus, 2019). For the full chemo-mechanical cycle of the F_1_-ATPase, it is likely that both mechanisms play a role. For example, the binding and release of the nucleotides are events associated with large conformational changes in the host domains, indicative of a power-stroke mechanism. The substep of the ATP hydrolysis in the pocket is energetically neutral, yet they also produce a small step of about 10° (Volkán-Kacsó and Marcus, 2018), which may indicate a ratcheting mechanism: the hydrolysis substep seems to gate the subsequent energetically downhill Pi release step from the neighboring pocket, thereby providing a directionality for the rotation. Hwang and Karplus also point out that the molecular mechanism of the elastic response of twisted or deformed protein structure in the motors has not been found in simulations. So, the emergent harmonic behavior seen in experimental single-molecule fluctuation data in various F_1_-ATPase species requires further investigation using Cryo-EM structure-based atomistic simulations. Warshel and co-workers have shown using free energy simulations that the detailed mechanism of the proton gradient-induced step-like kinetics of the Fo-ATPase is complex and involves the displacement of multiple key residues (Bai and Warshel, 2019). These mechanisms appear to have the qualities of both power stroke and Brownian ratchet-like unidirectional processes.

## Comparison of Kinetics and the Structural Behavior Inferred From Cryo-EM Studies

Unidirectional (counter clockwise when viewed from the site where the F_o_ would be) rotation of the F1-ATPase γ shaft in unconstrained (free) rotation experiments was first demonstrated using fluorescent filaments attached to the rotor shaft (Noji et al., 1997) and later confirmed in numerous single-molecule imaging experiment using beads and nanoparticles as probes. The unidirectionality was later suggested to be “encoded” in the structure of the stator ring subunits, when the conformational kinetics of rotorless F_1_-ATPase was imaged in high-speed AFM experiments (Uchihashi et al., 2011).

**Figure 5:**
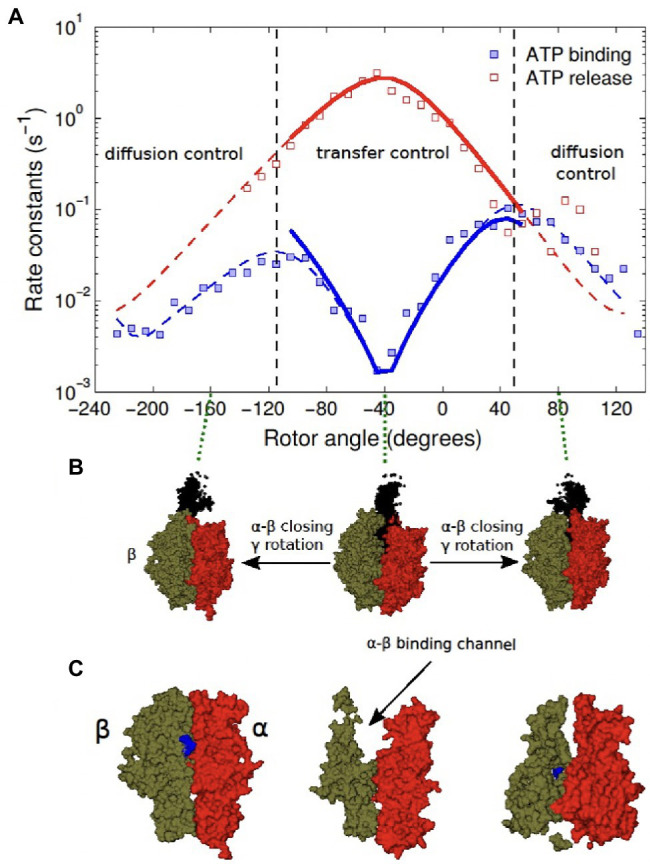
**(A)** Reported binding and release rate constants vs. controlled rotation angle for fluorescent ATP in the presence of Pi in solution. The reported uncorrected experimental data (squares) are compared with theoretical counterparts (solid lines) by calculating missed events and also correcting for an error due to replacing the time in the empty state T_0_ by the total time T. Dashed lines show a fit to the experimental data. **(B)** F_1_-ATPase structure at three different rotor angles with β subunits in green, α subunits in red and the γ subunit in black. **(C)** Cutaway of the three structures (PDB:1H8E) revealing the binding channel at the α − β interface and its narrowing as the rotor angle is changed. The figure was reproduced with permission from Volkán-Kacsó and Marcus (2017b).

To address the structural basis mechanism of the directed rotation in the F_1_-ATPase, we consider the X-ray crystallography (Braig et al., 2000; Menz et al., 2001) and more recent Cryo-EM structures (Guo and Rubinstein, 2018; Sobti et al., 2021). We first note that an interplay of molecule transfer and diffusion-controlled regimes was suggested in an earlier study (Volkán-Kacsó and Marcus, 2016) of the controlled rotation experiments (Adachi et al., 2012; Watanabe et al., 2012). In the study, a theory of the time-dependent kinetics is developed for the angle-dependent lifetimes of the observed nucleotide binding and release events. The system is at all times driven by an external torque and so it is out of equilibrium, which is reminiscent of earlier force-ramp pulling experiments (Hummer and Szabo, 2010). A turnover in the ATP-binding rate constants vs. rotor angle plot is seen in Figure 5A, as the closing of the binding channel continues beyond 80°.

It was suggested (Volkán-Kacsó and Marcus, 2016) that the ATP transits the channel at the α−β subunit interface (Figure 5C) by a transfer mechanism, after an initial docking of the ATP to the outside of the F_1_-ATPase. Features from X-Ray crystallography structures (Braig et al., 2000; Menz et al., 2001) supported this mechanism that relates the opening/closing of channels, rotation of the shaft and the entering/exiting of nucleotides.

**Figure 6:**
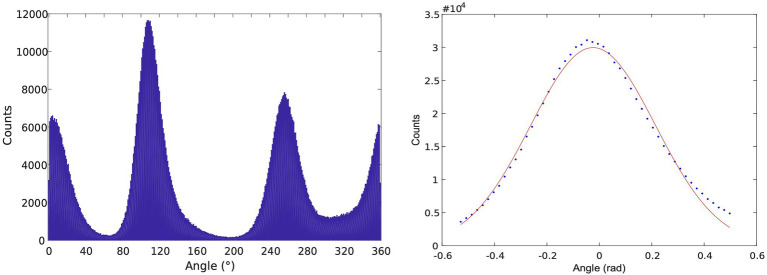
Left: Histogram of angular positions in the dwells from ThF1 rotation trajectories. Right: Consolidated histogram of all three subunits after selecting for “high-quality” dwells. More recently, a correction method was developed for treating the asymmetry between subunits using a tilting algorithm. The figure was reproduced from Volkán-Kacsó et al. (2019).

In the reaction scheme, the transfer reaction determines the angle dependence in the θ-region from about −45° to +45° where stalling and controlled rotation experiments overlap. In a transfer mechanism, a cleft closes upon the transfer of an ATP molecule into an “internal” binding pocket formed at the interface between neighboring α and β subunits. A cross section of the barrel structure of the stator ring and rotor shaft is shown in Figure 5 for an open and a closed cleft. In these X-ray crystallography structures, the binding channel is formed by the two subunits and it appears to close upon the binding of an ATP molecule. At angles outside of ± 50° or so, the binding channel leading to the binding pocket (Figure 6C) closes as the rotor is moved in the positive direction (Figure 6B). This inference was recently verified in Cryo-EM structures, which were assigned to dwells in the active kinetic cycle of the F_1_-ATPase motor (Guo and Rubinstein, 2018). This narrowing of the channel retards the diffusion process so that diffusion becomes the rate-limiting step during binding. The rotation of shaft, opening/closing of the channel, and displacement of the nucleotides are related as follows, using current structure and kinetic data:

1. Rotating the shaft either counterclockwise or clockwise starting from −40° induces a closing of the *β* subunit which accelerates binding. This behavior is consistent with the spontaneous forward rotation induced by binding of ATP in free rotation experiments occurring in a θ-range to the right of −40° (from 0 to 80°).
2. Rotating the *γ* rotor towards −40° either clockwise or counterclockwise induces an opening of the β subunit, which accelerates ADP (or ATP) release. This result is consistent with the spontaneous forward rotation induced by the release of ADP in free rotation experiments occurring in a θ-range to the left of −40° (from −20 to −40°).
3. When rotation induces an increased narrowing of the channel, diffusion becomes the bottleneck process and closing of the channel slows down both binding and release processes. So, approaching the most closed conformation (at +140°) in either clockwise or counterclockwise direction will slow down the binding and release of nucleotides in free rotation experiments.

The unidirectionality of the rotation then is due to the specific angular ranges where the rotation-driving (power-stroke) event occurs: binding of ATP in the −40 to 40° range drives the shaft *forward*, release of ADP (at another site) in the next 40° range dominates and drives the rotation *forward* (ADP release in the 280°–320° range in the other site induces closing and forward rotation, as seen in Figures 5C, 6). Finally, the remaining 40° rotation *forward* is presumably driven by Pi release and to a small extent the ATP hydrolysis step. We note that recently a role of the ϵ subunit was revealed in accelerating product ATP release when the motor rotates in the synthesis direction (Watanabe et al., 2018).

In a recent kinetic modeling by Dill and coworkers (Wagoner and Dill, 2019), a minimal two-state model was employed to discuss the question of efficiency vs. speed in the molecular motors. Their model could be extended by using a kinetic scheme in which the rate constants depend on a generalized structural slow coordinate, such as the angle of the rotor shaft or a hinge structure that opens and closes upon nucleotide binding.

## Relation Between Mechanical Motion and Chemistry

In the section “Use of data on ensemble studies to assist in predictions of single molecule measurements,” we discussed angle-dependent rate constants for individual steps. From them, occupation probability density profiles can also be calculated. In itself, the rate constants data do not actually explain the connection between the mechanical motion and the chemical reactions. Further insight into the latter is obtained from additional observations related to these rate constant data, by comparing population densities. It was found from studies where the typical total occupancy in the three sites was no larger than unity (Volkán-Kacsó and Marcus, 2017a) that there nevertheless was a correlation between the probability of exit of an ADP and the probability, in another subunit, of entrance of an ATP even though, as mentioned, the total occupation probability of the three sites was not greater than unity.

The probability of ATP binding early in the cycle was correlated with the probability density of an ADP escape some 240° later. It was inferred therefore that the correlation of the two events is inherent in the correlated structural behavior of the clefts from different subunits and did not require actual occupation of both clefts for the correlation to occur. So, the correlation between an ATP binding and ADP release at another site occurs more or less simultaneously. It implies that if computations with a correct force field are undertaken one does not require occupancy for there to be a correlation between the behavior opening (closing) of an ATP site and that closing (opening) of an ADP site. Both the γ shaft and the individual α and β subunits have asymmetric shapes and when they are in contact rolling over each other during the rotation: there is a correlated behavior whereby while one cleft is opening the other is closing. There is predicted in this way to be an approximate conservation of total unoccupied volume in the three empty clefts during the rotation.

We also note that comparing the various single-molecule experiments on F_1_-ATPase with the actual enzyme F_o_F_1_-ATP synthase the single-molecule experiment of the controlled rotation type resembles most closely the behavior of the actual F_o_F_1_ enzyme since both have a constant rotation rate, the one in the ATP synthase being discretized in the form of successive proton transfers to the c-ring members while the one in the single molecule experiment being continuous.

## Relation to Other Theoretical and Computational Work

A probabilistic approach to treat a discrete stepping model of the rotary motor F1-ATPase as observed using a microbead optical probe was pioneered by Oster and Wang (2000). In an early version of the model, all combinations of 64 possible kinetic states were considered, and a discrete numerical method was developed for solving the Fokker–Planck equations in an attempt to treat the full cycle of the chemo-mechanical coupling. In a theoretical work assuming a simpler, sequential kinetics, Junge and co-workers proposed an elastic mechanical coupling between the rotation of the F_O_ and the F_1_ (Panke et al., 2001). The latter resolved the puzzle of efficient motor function when the number of subunits in the $\alpha_{3}\beta_{3}$ barrel (F_1_ part) is not commensurate with that of subunits in the C-ring (F_O_ part).

Alternatively, a Langevin dynamics approach has the advantage of generating simulated trajectories, which can then be subjected to the same analysis as experimental single-molecule trajectories. Langevin simulations were used to simulate a discrete-state model with rotation angle-dependent switching potentials, modeled after single-molecule experiments (Watanabe et al., 2013). Langevin dynamics was also used with potentials derived from electrostatic free energy calculations based on atomistic structure (Mukherjee and Warshel, 2011).

Single-molecule imaging provides time-dependent observations of the motors at an individual motor level. They introduce observables that were not considered when traditional statistical thermodynamics was developed for ensembles that describe the bulk system. F_1_-ATPase proved to be a robust “model system” for the development of new stochastic tools suitable for single-molecule experiments. An example is “fluctuation theorems” used to extract steady-state observations from fluctuating single-molecule observation in the context of discrete-state models (Andrieux and Gaspard, 2006; Astumian, 2007), including experiments in which single molecules were driven out of equilibrium by time-dependent pulling forces (Hummer and Szabo, 2010) or torques (Volkán-Kacsó and Marcus, 2016).

Together with single-molecule experiments that revealed the concerted kinetics, the early theoretical works were the precursors of the elastic molecule transfer model of angle-dependent rates in the steps in the active chemo-mechanical pathway of F1-ATPase rotation. In practical applications, relating the stochastic behavior to experimentally observable trajectories requires an additional (painstaking) data analysis step. An approach used in our studies is to remain close to the experiments, in the present case performing the data analysis of fluctuating trajectories to extract experimentally observable quantities, such as histograms, velocities, and correlation functions from the single-molecule data (Volkán-Kacsó and Marcus, 2021). Then, using the theoretical models, the theoretical counterpart of these observable quantities was derived and used to predict or fit experimental single-molecule data.

The atomic resolution of the F_1_-ATPase structure in various nucleotide occupancy conditions and conformational states (Walker, 2013) triggered several studies using computer simulations. The cleavage reaction during hydrolysis in the tightly closed binding pocket was simulated using an embedded quantum-classical method (Dittrich et al., 2004). In these studies, the overall conformation of the host subunits was unchanged during the transition from ATP to a cleaved ADP and Pi. Even so, the coupling between chemistry and conformation was demonstrated: They found that an arginine “finger,” if in close proximity to the bond being ruptured, reduces the reaction free energy barrier, thereby accelerating the reaction. Conversely, in a conformation where the arginine is not close to the bond, the reaction proceeds an order of magnitude more slowly (Dittrich et al., 2004).

The relation of structure, as revealed by X-ray crystallography, and function, as revealed in rotation experiment, has been pursued also by atomistic computational methods. In an early study of the conformational change in the ring subunits leading to a rotation of the shaft, it was revealed by steered molecular dynamics simulations that the closure of the subunit hinges induced a 80/40 degree stepping rotation (Pu and Karplus, 2008). Simulations with accurate electrostatic modeling yielded a 2-dimensional free energy profile, concluding that a minimum energy path results in the 80/40 substeps (Mukherjee and Warshel, 2011). Large conformational changes of hinge-bending of individual β subunits leading to rotation of the γ shaft were studied with all-atom force-field free energy calculations (Ito et al., 2011). The free energy profiles calculated along interpolated structure between open and closed structures in the presence or absence of ATP yielded quadratic-like profiles which are consistent with the energy relation from equation (3) used in the elastic transfer theory for the ATP binding step in the kinetic scheme from Figure 3.

In the theoretical study that revealed a short-lived state in the transitions between subsequent dwells (Volkán-Kacsó et al., 2019), the state is predicted to be a three-occupancy state in which one of the sites will release an ADP molecule within about 10 ms. Large-scale simulations can be used to test this prediction of nucleotide release. The displacement of nucleotides and Pi in the binding pockets is coupled with conformational changes in the structure. Molecular dynamics simulations were used to estimate the diffusion constant for Pi diffusion in the channel (Hummer, 2005). The diffusion of the Pi molecule in unbiased MD simulations induced a significant conformational change in the host subunit (Okazaki and Hummer, 2013). The Pi release on the scheme from Figure 3. occurs after the release of ADP, and the work by Hummer’s team suggests that the protonation state of the Pi might play a key role in its delayed release: a doubly charged Pi, i.e., HPO_4_^2−^, was found to be tightly bound in the catalytic pocket, as opposed to a singly charged H_2_PO_4_^−^.

There is a rapidly growing body of structures resolved for various species of rotary motors, including the V-ATPase family. The experimental groups of Murata, Wilkens, and Rubinstein have reported in recent years structures relevant to the kinetic mechanism of V-ATPase (Arai et al., 2013; Oot et al., 2016; Ueno et al., 2018; Abbas et al., 2020). Given the similarity in the α_3_β_3_ rings of the F- and V-ATPases, it is conceivable that the mechanism of accelerated ADP release induced by the binding of ATP to another subunit was conserved over eons of evolution, even as their different functions arose, e.g., V-ATPases, as opposed to certain F-ATPases are not reversible, they are proton pumps that cannot synthesize ATP. The theoretical and computational study of a hypothetical universal mechanism for the allosteric nucleotide binding-release mechanism in rotary motors should be pursued.

## Author Contributions

SV-K and RM planned and performed the research and wrote the manuscript. All authors contributed to the article and approved the submitted version.

## Funding

This work was supported by the Office of the Naval Research and the James W. Glanville Foundation.

## Conflict of Interest

The authors declare that the research was conducted in the absence of any commercial or financial relationships that could be construed as a potential conflict of interest.

## Publisher’s Note

All claims expressed in this article are solely those of the authors and do not necessarily represent those of their affiliated organizations, or those of the publisher, the editors and the reviewers. Any product that may be evaluated in this article, or claim that may be made by its manufacturer, is not guaranteed or endorsed by the publisher.
